# PDLIM1: Structure, function and implication in cancer

**DOI:** 10.15698/cst2021.08.254

**Published:** 2021-07-26

**Authors:** Jian-Kang Zhou, Xin Fan, Jian Cheng, Wenrong Liu, Yong Peng

**Affiliations:** 1 Laboratory of Molecular Oncology, Frontiers Science Center for Disease-related Molecular Network, State Key Laboratory of Biotherapy and Cancer Center, West China Hospital, Sichuan University, Chengdu 610041, China.; 2 Department of Neurosurgery, West China Hospital, Sichuan University, Chengdu 610041, China.

**Keywords:** PDLIM1, CLP36, cytoskeleton, EMT, cancer progression

## Abstract

PDLIM1, a member of the PDZ-LIM family, is a cytoskeletal protein and functions as a platform to form distinct protein complexes, thus participating in multiple physiological processes such as cytoskeleton regulation and synapse formation. Emerging evidence demonstrates that PDLIM1 is dysregualted in a variety of tumors and plays essential roles in tumor initiation and progression. In this review, we summarize the structure and function of PDLIM1, as well as its important roles in human cancers.

## INTRODUCTION

PDLIM1 (PDZ and LIM domain protein 1), also known as CLP36, Elfin or CLIM1, is an important player in cytoskeletal organization, neuronal signaling and organ development through interacting with a variety of proteins, such as α-actinin, paladin, FHL1 and EGFR [1–5]. Four α-actinin genes (ACTN1-4) are embedded in human genome and their expression exhibits tissue-specific patterns. ACTN2 and ACTN3 are highly expressed in skeletal muscle and cardiac muscle sarcomere, respectively, whereas ACTN1 and ACTN4 are widely expressed in non-muscle cells [3, 6–9]. PDLIM1 forms a complex with α-actinin-1/4 in colonic epithelial cells and localizes to actin stress fibers [1, 6], while the interaction between PDLIM1 and α-actinin-2 was found in human myocardium [10]. What's more, in the dorsal root ganglion neurons PDLIM1 interacts with palladin to influence neurite outgrowth during sciatic nerve regeneration [2].

Elaborate regulation of PDLIM1 is crucial for maintaining homeostasis under physiological conditions. However, increasing evidence recently demonstrates that PDLIM1 is dysregulated in a variety of tumors, such as colorectal cancer (CRC) [11], hepatocellular carcinoma (HCC) [12], breast cancer [13], pancreatic cancer [14], glioma [15, 16] and chronic myelogenous leukaemia (CML) [17]. Moreover, PDLIM1 plays important roles in cell proliferation and metastasis during tumor initiation and progression. In this review, we summarize the structure and function of PDLIM1 and outline the research progress of PDLIM1 in human cancers.

## STRUCTURE OF PDLIM1 PROTEIN

PDLIM1 belongs to the actinin-associated LIM protein (ALP) subfamily of PDZ-LIM protein family [18]. The proteins of the ALP subfamily include ALP, reversion-induced LIM protein (RIL), Mystique and PDLIM1, which are characterized by a PDZ domain at the amino terminus and a LIM domain at the carboxyl terminus [18, 19].

**Figure 1:**
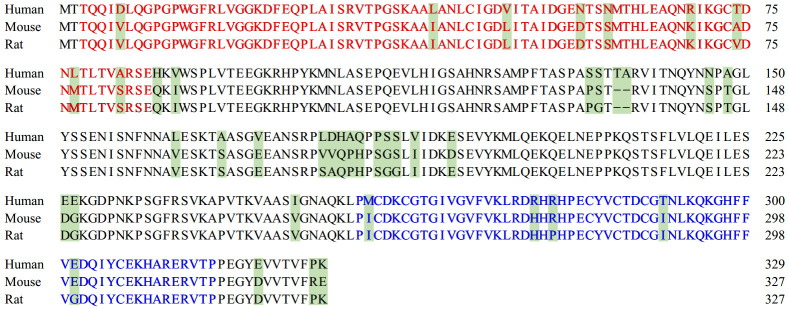
FIGURE 1: Comparison of the amino acid sequences of PDLIM1 between human, mouse and rat. The different amino acids are shaded. The red ones represent the PDZ domain, and the blue ones represent the LIM domain. UniProt Entry of PDLIM1: No. O00151 (Human), No. O70400 (Mouse) and No. P52944 (Rat).

PDLIM1 was originally cloned from normoxic rat hepatocytes in 1995. The rat PDLIM1 is encoded by 327 amino acids (AAs) and is highly homologous to many LIM domain proteins, particularly rat RIL [20, 21]. The overall homology of PDLIM1 to rat RIL was 45.1%, with 62% homology at the N-terminus (AA 1-89) and 50% homology at the C-terminus (AA 192-327). Subsequently, Kotaka *et al.* characterized the human PDLIM1 cDNA clone [22]. Since the ORF of this cDNA encodes a 36-kDa carboxyl terminal LIM domain protein with a PDZ domain at the amino terminal, which shares high homology to rat CLP36 (36 kDa carboxy-terminal LIM domain protein), the encoded protein was named human 36 kDa carboxy-terminal LIM domain protein (hCLIM1) [10, 22]. Human PDLIM1 is composed of 329 AAs and represents high conservation and homology in structure among species (**Figure 1**). The identity of its PDZ domain to human ALP is 55% and that of human RIL is 66%, while the LIM domain identity was 67% to ALP and 57% to RIL, respectively [1]. In view of the high sequence homology between PDLIM1 and other proteins in the ALP subfamily, it is not surprising that the function of PDLIM1 in cells can be partially rescued by proteins such as RIL [23], whereas in some cases it cannot be compensated [13]. Therefore, the specific mechanisms of PDLIM1 and other ALP family proteins are worthy of in-depth study.

**Figure 2:**
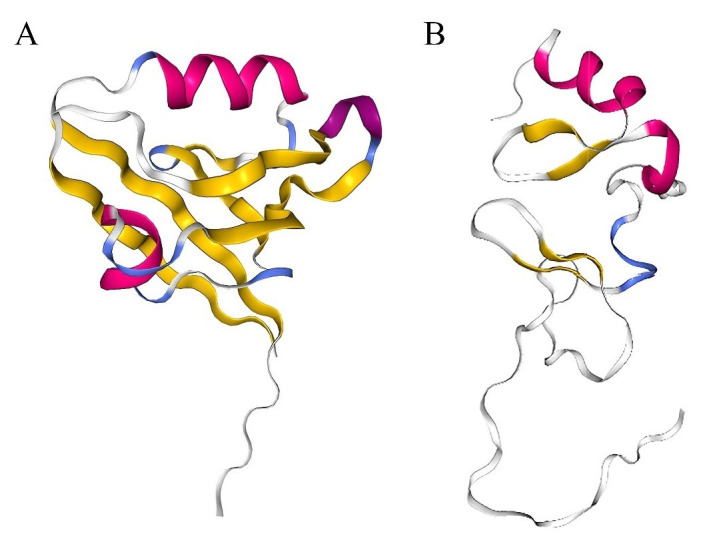
FIGURE 2: Crystal structure of human PDZ domain and LIM domain. **(A)** The PDZ domain consists of α-helix (pink), 3/10 helix (purple), β strand (yellow), β turn (blue), and coil (white). PDB ID: 2PKT. **(B)** The LIM domain consists of α-helix (pink), β strand (yellow), β turn (blue), and coil (white). PDB ID: 1X62.

Since the protein structure of PDLIM1 has not been fully analyzed, expounding its two domains, PDZ and LIM, may be helpful to understand the role of PDLIM1. The PDZ domain (AA 3-85 of PDLIM1, PDB ID: 2PKT), whose name is derived from PSD-95, DLG and ZO-1 proteins that were first discovered to contain this domain, consists of antiparallel β chains and α helices [24–26] (**Figure 2A**). The PDZ domain is highly conserved and widely present in the proteins of bacteria, fruit flies, plants and animals. As an important domain that mediates protein interactions, it provides protein binding sites and can mediate the formation of multiple protein complexes, including membrane-associated proteins, cytoplasmic signaling proteins, and cytoskeletal proteins [27, 28]. The proteins that have been reported to bind to the PDZ domain of PDLIM1 include α-actinin, p75^NTR^ and β-catenin/E-cadherin complex [6, 11, 13, 15]. Another domain of PDLIM1, the LIM domain (AA 258-317 of PDLIM1, PDB ID: 1X62), was named after its first recognition in Lin11, Isl-1 and Mec-3 [29–31]. The LIM domain consists of two zinc finger domains exhibiting a consistent Cys-rich sequence (Cys-X_2_-Cys-X_17±1_-His-X_2_-Cys)-X_2_-(Cys-X_2_-Cys-X_17±1_-Cys-X_2_-His/Asp/Cys) (X represents any AA) (**Figure 2B**) [32–34]. The LIM domain is responsible for the interaction between PDLIM1 and proteins such as kinases and actin cytoskeletal components [10, 35]. Generally, through PDZ and LIM domains, PDLIM1 interacts with α-actinin, palladin, and kinases, as well as serves as a scaffold to promote the formation of protein complexes, thereby regulating signal pathways and affecting cell activity [36, 37].

## BIOLOGICAL FUNCTION OF PDLIM1

PDLIM1 is widely distributed in heart, lung, liver, and spleen tissues [18, 38]. By interacting with various proteins in different tissues, PDLIM1 participates in the regulation of multiple signal transduction pathways and exhibits tissue-specific functions.

As a cytoskeleton-associated protein, PDLIM1 is involved in the regulation of actin cytoskeleton organization. The dynamic regulation of the assembly and disassembly of stress fibers is essential for cytoskeleton-dependent functions such as morphological changes and migration [39, 40]. Previous studies have shown that inhibition of PDLIM1 impairs the assembly of focal adhesion and the formation of stress fibers in BeWo cells, and these effects can be reversed by expressing exogenous PDLIM1 (CLP36), rather than by expressing its mutant form of lacking PDZ or LIM domains, indicating the important role of PDZ and LIM domains in maintaining cytoskeleton-dependent functions [41]. Moreover, PDLIM1-deficienct fibroblasts exhibit a reduction in stress fiber formation and a significant increase in directional and random migration, and these abnormalities caused by PDLIM1 deletions can be compensated by RIL protein [23]. In addition to affecting the formation and dynamics of stress fibers, PDLIM1 can also act as an adapter molecule that recruits signal molecules to stress fibers. For example, Vallenius *et al.* confirmed the LIM domain-dependent interaction between PDLIM1 and CLP-36 interacting kinase 1 (Clik1) by yeast two-hybrid analysis. This interaction with PDLIM1 makes Clik1, which is originally located in the nucleus, dramatically relocate to actin stress fibers [35]. However, whether this relocation leads to changes in the function of Clik1 remains to be explored. Furthermore, a recent study showed that PDLIM1 resides in the actin-rich structure induced by invasive and adherent bacterial pathogens, such as *Listeria monocytogenes* and enteropathogenic *Escherichia coli* (EPEC), but the specific mechanism still needs further study [42].

The correct assembly of the cytoskeleton regulated by PDLIM1 is essential for the normal function of Sertoli cells and the ordered spermatid differentiation during spermiogenesis [43, 44]. Ectoplasmic specialization (ES) is a testis-unique anchoring junction and essential for Sertoli-germ cell communication and successful spermatogenesis [45]. Under normal circumstances, redundant PDLIM1 is degraded by the autophagic lysosomal pathway in Sertoli cells to promote proper assembly of ES and maintain an appropriate cytoskeletal network during sperm formation. However, when autophagy is destroyed by knocking out autophagy-related proteins ATG7 or ATG5, PDLIM1 is accumulated in the cytoplasm of Sertoli cells and disrupts the F-actin hoops of apical ES and related microtubule-based structures in the seminiferous epithelium, leading to disordered cytoskeleton structures and broken ES assembly, thus affecting Sertoli-germ cell communication [43]. Similarly, knockout of *Atg7* in spermatids blocks autophagy and upregulates PDLIM1 in the cytoplasm, resulting in the formation of stress fibers-like structures and the disruption of spermatozoa flagella assembly and spermatid differentiation, eventually leading to sterility [44].

It is reported that PDLIM1 is expressed in the sensory ganglia of adult rats, but not in the central nervous system, and its expression is upregulated in peripheral sensory neurons and motor neurons after sciatic nerve transection, suggesting that PDLIM1 may play a certain role in the regulation of neurite growth [46]. In primary dorsal root ganglion (DRG) neurons and undifferentiated PC12 cells, PDLIM1 is distributed in the cell body and neurites and is highly enriched in the growth cone. Functionally, overexpression of the PDLIM1 PDZ domain inhibits the outgrowth of neurite. Instead, knockdown of PDLIM1 in PC12 cells with shRNA altered cell morphology and activated growth cone movements, resulting in increased length and number of neurites. Likewise, inhibition of PDLIM1 in primary DRG neurons increased the growth rate of neurite cells [46]. These results demonstrate that PDLIM1 plays an important role in controlling neurite outgrowth.

Furthermore, PDLIM1 is also involved in the regulation of signaling pathways. The transcription factor NF-κB, a class of dimer proteins consisting of p65 (RelA), NF-κB1 (p50/p105), NF-κB2 (p52/p100), c-Rel and RelB, is usually inactivated through binding to its inhibitor protein IκB [47]. When stimulated by Toll-like receptors (TLR) signaling, IκB can be phosphorylated by the activated IKK complex and is eventually degraded by the ubiquitin ligase machinery. Thus, the freed NF-κB dimer is translocated into the nucleus, where it initiates the transcription of target genes related to the inflammatory reaction [48, 49]. PDLIM1 was reported to be an important factor in modulating p65 nuclear translocation, thereby participating in the regulation of the NF-κB pathway. In dendritic cells, p65 was shown to bind to PDLIM1 and thus be retained in the cytoplasm, thereby reducing its nuclear translocation and NF-κB-mediated inflammatory signaling. Notably, the inhibition of NF-κB signaling by PDLIM1 is independent of IκBα, but dependent on α-actinin-4, the binding protein of PDLIM1, because knocking down α-actinin-4 reversed the inhibition of p65 nuclear translocation mediated by PDLIM1. Besides, deficiency of PDLIM1 in mice leads to the elevated levels of nuclear p65 protein, and thus promotes the production of proinflammatory cytokines and chemokines (such as IL-6, IL-12, TNF*α*, IL-18, CXCL2 and CXCL10), indicating that PDLIM1 negatively regulates NF-κB-mediated inflammation in innate immune response [50]. Therefore, PDLIM1-mediated inhibition of the NF-κB pathway may serve as a new strategy for the treatment of inflammatory diseases.

## PDLIM1 AND CANCER PROGRESSION

PDLIM1 plays an important role in maintaining certain homeostasis of cells. The dysregulation of PDLIM1 usually manifests as its abnormal expression, which affects the interaction with its binding proteins and the related signal pathways (such as NF-κB, Wnt/β-catenin and Hippo), leading to the occurrence and development of many diseases including cancer.

### PDLIMI and CRC

CRC is the third most common cancer worldwide, accounting for approximately 10% of all new cases and deaths each year [51]. The major cause of death in CRC patients is metastasis, which can be promoted by epithelial-mesenchymal transition (EMT) [52, 53]. EMT is a biological process in which epithelial cells are transformed into mesenchymal cells under specific conditions, characterized by the down-regulation of epithelial factors (E-cadherin, claudins) and activation of mesenchymal factors (N-cadherin, vimentin) [54]. In CRC, deregulation of the canonical Wnt/β-catenin signaling pathway increases SNAIL, leading to the reduction of E-cadherin and thus promoting EMT [55]. Moreover, β-catenin and E-cadherin can form an E-cadherin/β-catenin complex, which can maintain tight junctions between cells and prevent cell invasion and metastasis. Factors such as TGF-β can dissociate and translocate β-catenin from the E-cadherin/β-catenin to the nucleus, thereby activating the Wnt pathway and inducing EMT [56–58].

**Figure 3:**
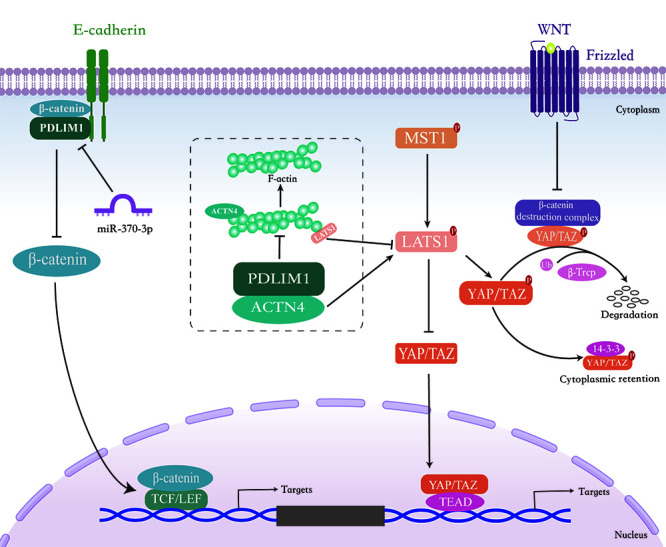
FIGURE 3: Schematic model of PDLIM1-mediated cancer progression. PDLIM1 expression is down-regulated in CRC cells, thus reducing the binding of β-catenin/E-cadherin complex and subsequent β-catenin nuclear translocation to initiate EMT and metastatic process. In HCC cells, loss of PDLIM1 leads to the formation of excessive F-actin, further inducing LATS1 dephosphorylation, inactivating the Hippo pathway and promoting HCC metastasis. Moreover, PDLIM1 expression is regulated by miRNAs such as miR-370-3p, subsequently affecting downstream pathways.

Chen *et al.* found that PDLIM1 was down-regulated in CRC tissues compared to peritumoral tissues due to the hypermethylation of the PDLIM1 gene promoter in CRC tissues. Importantly, loss of PDLIM1 promotes the invasion and metastasis of CRC cells *in vitro* and *in vivo*. By contrast, enhanced PDLIM1 inhibits CRC cell invasion and metastasis. Moreover, the deletion of PDLIM1 down-regulates the expression of epithelial factors and promotes the expression of mesenchymal factors, indicating that PDLIM1 promotes the invasion and metastasis of CRC cells by affecting the EMT process. Mechanistically, down-regulation of PDLIM1 in CRC cells reduces the binding of β-catenin and E-cadherin, leading to the β-catenin nuclear translocation to initiate EMT and metastatic process in CRC cells, suggesting that PDLIM1 is involved in the regulation of cell invasion and migration and exerts a tumor suppressor function in CRC. Furthermore, repression of PDLIM1 in CRC tissues predicts poor survival in CRC patients [11]. Taken together, PDLIM1 can interact with E-cadherin/β-catenin complex, thereby suppressing the transcriptional activity of β-catenin and inhibiting the occurrence of EMT in CRC (**Figure 3**). Therefore, PDLIM1 may serve potentially as a marker of tumor aggressiveness and as a predictor of survival in CRC patients.

Intriguingly, Rai *et al*. reported that PDLIM1 in neonatal human foreskin fibroblasts (neoHFFs) can act as a pro-invasive regulator of membrane protrusion. PDLIM1 is upregulated in neoHFFs activated by late-stage colorectal cancer-exosomes (exosomes derived from SW620 CRC cells), allowing cells to acquire the ability to invade through the extracellular matrix [59]. Accordingly, the function of PDLIM1 in CRC may be highly context-dependent, which provides more perspectives for the study of PDLIM1.

### PDLIM1 and HCC

The Hippo pathway is an evolutionarily conserved tumor-suppressor signaling pathway, which plays an essential role in a variety of tumors including HCC [60, 61]. The core components of the Hippo pathway include MST1/2, LATS1/2, YAP, and downstream target genes. When Hippo signaling is on, activated MST1/2 kinases can phosphorylate and activate its substrate LATS1/2, which in turn phosphorylates the effector YAP. The phosphorylated YAP are retained in the cytoplasm due to the interaction with the 14-3-3 protein and further binds to the β-Catenin destruction complex to trigger β-TrCP-mediated degradation. However, when Hippo signaling is off, YAP is stabilized and translocated to the nucleus, and combines with transcriptional activators to initiate transcription of downstream genes such as CTGF, CYR61, thereby promoting tumor progression [62].

Our group reported that PDLIM1 expression is significantly decreased in metastatic HCC tissues compared to non-metastatic tumors, suggesting that PDLIM1 may play an inhibitory role during HCC metastasis [12]. Functional studies showed that knockdown of PDLIM1 in HCC cells can induce EMT and promote metastasis *in vitro* and *in vivo*, while overexpression of PDLIM1 shows the opposite phenotype. To examine the associated protein of PDLIM1 in HCC, we performed immunoprecipitation and mass spectrometric analysis, and identified that PDLIM1 interacts with the cytoskeleton cross-linking protein ACTN4. Specifically, PDLIM1 competitively binds to ACTN4 through Asn145 (N145), thereby weakening the interaction between ACTN4 and F-actin and preventing the overgrowth of F-actin. Therefore, loss of PDLIM1 in HCC cells leads to the formation of excessive F-actin, which further induces LATS1 dephosphorylation, inactivates the Hippo pathway and promotes HCC metastasis (**Figure 3**). Furthermore, low expression of PDLIM1 is associated with poor prognosis in HCC patients [12]. Therefore, our findings dissect the role and the underlying mechanism of PDLIM1 in HCC metastasis, and also highlight its prognostic potential in HCC.

### PDLIM1 and breast cancer

Breast cancer accounts for 30% of female malignancies and has a mortality rate of 15% worldwide [51]. Pitteri *et al*. reported that PDLIM1 expression is increased during breast cancer progression, suggesting that PDLIM1 may promote breast cancer development [63]. Liu *et al*. further confirmed that PDLIM1 is a pro-metastatic factor in breast cancer and enhances the invasion and metastasis of breast cancer cells through α-actinin-Cdc42 signaling pathway. PDLIM1 knockdown by RNAi in MDA-MB-231 cells significantly represses metastasis *in vitro* and *in vivo*, while overexpression of PDLIM1 exhibits opposite phenotypes. Importantly, PDLIM1 combines with α-actinin-4 to form a PDLIM1-α-actinin complex, thereby promoting the activation of Cdc42, which is a key factor in regulating cell polarity and migration of breast cancer cells [13, 64]. Notably, the PDZ domain is responsible for the binding of PDLIM1 to α-actinin in breast cancer cells. Disruption of this interaction by deleting the PDZ domain eliminates PDLIM1-mediated cell migration, further demonstrating the essential role of PDLIM1-α-actinin complex in breast cancer migration and invasion. Based on these findings, targeting PDLIM1-α-actinin-Cdc42 axis may have a potential for inhibiting breast cancer metastasis and is worthy of further investigation.

### PDLIM1 and pancreatic cancer

Pancreatic cancer is a highly malignant digestive tract tumor, with a five-year survival rate of only 9% due to the difficulty to diagnose and treat [51]. The identification of tumor antigens provides the possibility for early diagnosis and targeted therapy of patients with pancreatic cancer. Dr. Hong analyzed the sera of 36 patients with pancreatic cancer and 68 sera from healthy donors and patients with other cancers to identify proteins that specifically induce humoral responses in pancreatic cancer patients. Proteins extracted from the human pancreatic cancer BxPC-3 cells were separated by 2-Dimensional polyacrylamide gel electrophoresis (2-D PAGE), and then transferred to the PVDF membrane. The membranes were incubated with the collected sera individually to detect the antibodies against BxPC3 proteins, and a protein of approximately 36kDa showed reactivity with sera from 38.9% patients of pancreatic cancer and 4.4% of the other two groups, suggesting its pancreatic cancer specificity. Mass spectrometric analysis was performed to confirm that the protein was PDLIM1 [14]. The pancreas/ampullary adenocarcinoma tissue array further proved the immunoreactivity of PDLIM1 against pancreatic cancer. In addition, PDLIM1 RNA levels were significantly higher in pancreatic cancer cell lines compared to colon, lung, and ovarian cancer cell lines, and PDLIM1 was located in both cell membrane and cytoplasm, suggesting that PDLIM1 may be a tumor autoantigen in pancreatic cancer. However, the role of PDLIM1 in pancreatic cancer still needs further research, which may help to explore the potential of PDLIM1 as an early diagnostic marker or drug target for pancreatic cancer.

### PDLIM1 and glioma

The p75 neurotrophin receptor (p75^NTR^, also known as CD271) is a key molecule that drives tumorigenesis, invasion and metastasis in glioma [65, 66]. Ahn *et al*. demonstrated PKA inhibition reduced the phosphorylation of p75^NTR^ at S303 and thus attenuated p75^NTR^-mediated glioma invasion, indicating that PKA-induced p75^NTR^ phosphorylation at S303 is required for glioma invasion. Intriguingly, a novel phosphorylation at S425 that located within the C-terminal PDZ binding motif of p75^NTR^ was detected by nano-liquid chromatography–tandem mass spectrometry in cells expressing p75^NTR^. Further experiments proved that the deletion or mutation of the PDZ binding motif resulted in the elimination of p75^NTR^-mediated glioma invasion, suggesting that p75^NTR^ and its interacting PDZ domain-containing protein may work together to regulate glioma invasion. Therefore, the p75^NTR^-interacting protein containing the PDZ domain was identified by mass spectrometry, and it was found that PDLIM1 may be a potential binding protein of p75^NTR^. Immunoprecipitation and *in vitro* pull-down experiments further demonstrated the direct interaction between PDLIM1 and the C-terminal cytoplasmic domain of p75^NTR^, and this interaction was reduced when S425 of p75^NTR^ was phosphorylated, proving that S425 is a key residue to mediate the interaction between PDLIM1 and p75^NTR^. Moreover, knockdown of PDLIM1 by shRNA abolished p75^NTR^-mediated glioma invasion *in vitro* and *in vivo* [15, 16]. Overall, the interaction of p75^NTR^ and PDLIM1, which depends on the unphosphorylated state of S425, mediates the invasion of glioma. Consequently, disruption of the interaction between the PDZ domain of PDLIM1 and p75^NTR^ may provide potential therapeutic strategies for patients with glioma.

### PDLIM1 and CML

CML is a hematopoietic malignancy derived from the abnormal proliferation of bone marrow hematopoietic stem cells, and accounts for 15% of new cases of leukemias in adults [67, 68]. MicroRNA, a class of small non-coding RNA that play essential roles in CML progression [69–71]. Recently, Li *et al*. reported that miR-370-3p can suppress the proliferation and induce apoptosis of CML cells by targeting PDLIM1 [17]. The expression of miR-370-3p was found significantly reduced in the peripheral blood mononuclear cells (PBMC) from CML patients. Functional studies indicate that miR-370-3p inhibits proliferation and promotes apoptosis of CML cells. Further research revealed that miR-370-3p can directly bind to the 3'-untranslated region of PDLIM1, proving that PDLIM1 is a target gene of miR-370-3p. In view of the role in promoting proliferation and suppressing apoptosis, PDLIM1 was found to be an oncogene in CML, and the overexpression of PDLIM1 weakened the tumor suppressive role of miR-370-3p in CML, indicating that miR-370-3p functions partially depends on the repression of PDLIM1. Moreover, the author proved that miR-370-3p inhibits Wnt/β-catenin signaling by targeting PDLIM1, thereby suppressing CML cell proliferation and inducing apoptosis. Collectively, these results demonstrate the vital role of miR-370-3p-PDLIM1-Wnt/β-catenin signaling axis in the progression of CML (**Figure 3**). Therefore, the development of drugs targeting the miR-370-3p-PDLIM1-Wnt/β-catenin signaling axis, combined with other strategies, may be helpful to improve the clinical treatment of CML.

## CONCLUDING REMARKS

PDLIM1 is a highly conserved cytoskeleton protein that belongs to the PDZ and LIM protein family. Under normal circumstances, PDLIM1 participates in maintaining homeostasis within cells. In cancer, however, the dysregulation of PDLIM1 can lead to abnormalities in a series of signaling pathways. Through interacting with actin filaments and other proteins, PDLIM1 can regulate the proliferation, metastasis and survival of malignant cells, and ultimately affect the progression of cancers. Therefore, more research on the function of PDLIM1 is helpful for the diagnosis and treatment of tumors in the clinic.

Tumor cells acquire migration and invasion capabilities through EMT, which refers to dynamic reorganization of the cytoskeleton. As a cytoskeleton protein, PDLIM1 is involved in the EMT process in a variety of tumors. Interestingly, PDLIM1 displays remarkable tissue-specific roles across cancers. It can inhibit the metastasis of CRC [11] and HCC [12], but promote the development of various cancers such as breast cancer [13] and CML [17]. It is well known that the process of tumorigenesis and development involves the combined action of multiple genes and cellular signaling pathways. Therefore, it seems reasonable to speculate that the tissue-specific characteristics of PDLIM1 may depend on the difference in its interacting proteins and regulatory signaling pathways in different cancer types. Although progress has been made in characterizing PDLIM1, the cellular and molecular mechanisms of PDLIM1 and the corresponding downstream signaling pathways in cancer still need further research, which may help deepen the understanding of cancer progression and provide new strategies for the optimization of targeted therapy or combined therapy in the clinic.

## Abbreviatons:

AA: – amino acid;
ACTN: – α-actinin;
ALP: – actinin-associated LIM protein;
Clik1: – CLP-36 interacting kinase 1;
CLP36: – carboxyl terminal LIM domain protein of 36 kDa;
CML: – chronic myelogenous leukaemia;
CRC: – colorectal cancer;
DRG: – dorsal root ganglion;
EMT: – epithelial-mesenchymal transition;
ES: – ectoplasmic specialization;
HCC: – hepatocellular carcinoma;
neoHFF: – neonatal human foreskin fibroblasts;
PDLIM1: – PDZ and LIM domain protein 1;
RIL: – reversion-induced LIM protein.
